# Supplementary material for: The Systems Biology Research Tool: evolvable open-source software
Source: BMC Syst Biol. 2008 Jun 29;2:55. doi: 10.1186/1752-0509-2-55 (PMC2446383; doi:10.1186/1752-0509-2-55)
Supplement: Additional file 1 — SBRT Archive. An archive of the current version of the Systems Biology Research Tool. [file 1752-0509-2-55-S1.zip › sbrt-1.4.0/doc/developers_guide/api/index-files/index-1.html]

A-Index


|  |  |  |
| --- | --- | --- |
| **Overview** \| Package \| Class \| Use \| **Tree** \| **Deprecated** \| **Index** \| **Help** | |  |
| PREV LETTER   **NEXT LETTER** | **FRAMES**    **NO FRAMES**     **All Classes** |  |


A B C D E F G H I J K L M N O P Q R S T U V W X Y Z 

---


## **A**

**AbstractCvComputer** - Class in sbrt.kernel.fba.comp: This abstract class is a skeleton implementation of `FluxCvComputer`. **AbstractCvComputer(IrrevRxnFluxome)** - Constructor for class sbrt.kernel.fba.comp.AbstractCvComputer: Constructs a new constraint-variation computer. **AbstractFluxIntervalComputer** - Class in sbrt.kernel.fba.comp: This abstract class is a skeleton implemenation of `FluxIntervalComputer`. **AbstractFluxIntervalComputer(FbaOptComputer)** - Constructor for class sbrt.kernel.fba.comp.AbstractFluxIntervalComputer: Constructs a new flux interval computer with the default zero cutoff. **AbstractFluxIntervalComputer(FbaOptComputer, double)** - Constructor for class sbrt.kernel.fba.comp.AbstractFluxIntervalComputer: Constructs a new flux interval computer with the provided zero cutoff. **AbstractLinearComb**<V> - Class in sbrt.kernel.math: This abstract class is a skeleton implementation of `LinearComb`. **AbstractLinearComb(double, double)** - Constructor for class sbrt.kernel.math.AbstractLinearComb: Constructs a new linear combination with the provided constant and zero cutoff. **AbstractLinearComb(double)** - Constructor for class sbrt.kernel.math.AbstractLinearComb: Constructs a new linear combination with the provided constant. **AbstractLinearComb.Term**<V> - Class in sbrt.kernel.math: This class is a skeleton implementation of `LinearComb.Term`. **AbstractLinearComb.Term(double, V)** - Constructor for class sbrt.kernel.math.AbstractLinearComb.Term: Constructs a new linear combination term with the provided coefficient and variable. **AbstractLinearComb.Term(V, double)** - Constructor for class sbrt.kernel.math.AbstractLinearComb.Term: Constructs a new linear combination term with the provided coefficient and variable. **AbstractLinearComb.Term(V)** - Constructor for class sbrt.kernel.math.AbstractLinearComb.Term: Constructs a new linear combination term with the provided variable. **AbstractLinkedHashSetFormat**<E> - Class in sbrt.shell.text: This abstract class is a skeleton implementation of `LinkedHashSetFormat`. **AbstractLinkedHashSetFormat(String, Pattern, SimpleFormat<E>)** - Constructor for class sbrt.shell.text.AbstractLinkedHashSetFormat: Constructs a new `LinkedHashSet` format from the provided arguments. **AbstractLinkedHashSetFormat(String, Pattern, Formatter<E>, Parser<E>)** - Constructor for class sbrt.shell.text.AbstractLinkedHashSetFormat: Constructs a new `LinkedHashSet` format from the provided arguments. **AbstractListFormat**<E> - Class in sbrt.shell.text: This abstract class is a skeleton implementation of `ListFormat`. **AbstractListFormat(String, Pattern, SimpleFormat<E>)** - Constructor for class sbrt.shell.text.AbstractListFormat: Constructs a new list format from the provided arguments. **AbstractListFormat(String, Pattern, Formatter<E>, Parser<E>)** - Constructor for class sbrt.shell.text.AbstractListFormat: Constructs a new list format from the provided arguments. **AbstractMapEntryFormat**<K,V> - Class in sbrt.shell.text: This abstract class is a skeleton implementation of `MapEntryFormat`. **AbstractMapEntryFormat(String, String, String)** - Constructor for class sbrt.shell.text.AbstractMapEntryFormat: Constructs a new map entry format from the provided arguments. **AbstractMapEntryFormat(String)** - Constructor for class sbrt.shell.text.AbstractMapEntryFormat: Constructs a new map entry format using the provided delimiter and zero-length strings as left and right paddings. **AbstractMapFormat**<K,V> - Class in sbrt.shell.text: This abstract class is a skeleton implementation of `MapFormat`. **AbstractMapFormat()** - Constructor for class sbrt.shell.text.AbstractMapFormat: **AbstractMapInputFile**<K,V> - Class in sbrt.shell.io: This abstract class is a skeleton implemenation of text-based map input files. **AbstractMapInputFile(String, FileFormat, Set<? extends K>, MapFormat<K, V>)** - Constructor for class sbrt.shell.io.AbstractMapInputFile: Constructs a new map input file from the provided arguments. **AbstractMapInputFile(String, FileFormat, MapFormat<K, V>)** - Constructor for class sbrt.shell.io.AbstractMapInputFile: Constructs a new map input file from the provided arguments. **AbstractMapOutputFile**<K,V> - Class in sbrt.shell.io: This abstract class is a skeleton implementation of `MapTextOutputFile`. **AbstractMapOutputFile(String, FileFormat, Set<? extends K>, MapFormat<K, V>)** - Constructor for class sbrt.shell.io.AbstractMapOutputFile: Constructs a new map output file from the provided arguments. **AbstractMapOutputFile(String, FileFormat, String, MapFormat<K, V>)** - Constructor for class sbrt.shell.io.AbstractMapOutputFile: Constructs a new map output file from the provided arguments. **AbstractMapOutputFile(String, FileFormat, MapFormat<K, V>)** - Constructor for class sbrt.shell.io.AbstractMapOutputFile: Constructs a new vector output file from the provided arguments. **AbstractMdMatrix**<R,C> - Class in sbrt.kernel.math.algebra: This abstract class is a skeleton implementation of `DoubleMatrixBuilder`. **AbstractMdMatrix()** - Constructor for class sbrt.kernel.math.algebra.AbstractMdMatrix: **AbstractPolynomial**<V> - Class in sbrt.kernel.math: This abstract class is a skeleton implementation of `Polynomial`. **AbstractPolynomial(double)** - Constructor for class sbrt.kernel.math.AbstractPolynomial: Constructs a new polynomial with the provided constant. **AbstractProcessManager** - Class in sbrt.shell.mng: This class is a skeleton implemenation of `KernelProcessManager`. **AbstractProcessManager()** - Constructor for class sbrt.shell.mng.AbstractProcessManager: **AbstractProgressMonitor** - Class in sbrt.kernel.util: This abstract class is a skeleton implementation of `ProgressMonitor`. **AbstractProgressMonitor()** - Constructor for class sbrt.kernel.util.AbstractProgressMonitor: Constructs a new progress monitor. **AbstractResultsManager**<R> - Class in sbrt.kernel.io: This class is a skeleton implemenation of `ResultsManager`. **AbstractResultsManager()** - Constructor for class sbrt.kernel.io.AbstractResultsManager: Constructs a new results manager. **AbstractRxn** - Class in sbrt.kernel.fba: This class is a skeleton implementation of ChemicalRxn. **AbstractRxn(LinearComb<String>, LinearComb<String>)** - Constructor for class sbrt.kernel.fba.AbstractRxn: Constructs a new chemical reaction from the provided reactants and products with the given name. **AbstractSbrtFile** - Class in sbrt.shell.io: This class provides a helper method for SBRT files. **AbstractSbrtFile()** - Constructor for class sbrt.shell.io.AbstractSbrtFile: **AbstractSmdMatrix**<R,C> - Class in sbrt.kernel.math.algebra: This abstract class is a skeleton implementation of `SmdMatrix`. **AbstractSmdMatrix()** - Constructor for class sbrt.kernel.math.algebra.AbstractSmdMatrix: **actionPerformed(ActionEvent)** - Method in class sbrt.shell.gui.SbrtGui: Responds to the provided action event. **actionPerformed(ActionEvent)** - Method in class sbrt.shell.gui.TextPaneGui: Responds to the provided action event. **active()** - Method in class sbrt.kernel.fba.proc.FluxActivityProcess.Results: Returns the number of active fluxes. **ACTIVE** - Static variable in class sbrt.shell.mng.fba.RandomConstraintsManager: The keyword used to denote the active constraint. **ACTIVITY\_COUNT\_FILE\_NAME** - Static variable in class sbrt.shell.mng.fba.FluxActivityManager: The keyword used to denote the name of the activity count output file. **ACTIVITY\_VECTOR\_FILE\_NAME** - Static variable in class sbrt.shell.mng.fba.FluxActivityManager: The keyword used to denote the name of the activity vector output file. **add(double)** - Method in class sbrt.kernel.math.HashLinearComb: Adds, in the mathematical sense, the provided constant to this linear combination. **add(LinearComb<? extends V>)** - Method in class sbrt.kernel.math.HashLinearComb: Adds, in the mathematical sense, the provided linear combination to this linear combination. **add(LinearComb<? extends V>)** - Method in interface sbrt.kernel.math.LinearComb: Adds, in the mathematical sense, the provided linear combination to this linear combination. **add(double)** - Method in interface sbrt.kernel.math.LinearComb: Adds, in the mathematical sense, the provided constant to this linear combination. **add(E)** - Method in class sbrt.kernel.util.UnmodifiableCollection: See Collection.add(Object). **add(E)** - Method in class sbrt.kernel.util.UnmodifiableList: See List.add(Object). **add(int, E)** - Method in class sbrt.kernel.util.UnmodifiableList: See List.add(int, Object). **add(E)** - Method in class sbrt.kernel.util.UnmodifiableSet: See Set.add(Object). **addAll(Collection<? extends E>)** - Method in class sbrt.kernel.util.UnmodifiableCollection: See Collection.addAll(Collection). **addAll(Collection<? extends E>)** - Method in class sbrt.kernel.util.UnmodifiableList: See List.addAll(Collection). **addAll(int, Collection<? extends E>)** - Method in class sbrt.kernel.util.UnmodifiableList: See List.addAll(int, Collection). **addAll(Collection<? extends E>)** - Method in class sbrt.kernel.util.UnmodifiableSet: See Set.addAll(Collection). **addData(S)** - Method in class sbrt.kernel.io.ListResultsManager: Adds the provided results to this results manager. **addData(S)** - Method in interface sbrt.kernel.io.ResultsManager: Adds data to this results manager. **addData(S)** - Method in class sbrt.kernel.io.SingleRm: Adds the provided results to this results manager. **addData(Map<? extends K, ? extends V>)** - Method in class sbrt.shell.io.ComoFile: Writes the appropriate values of the provided vector to this file. **addData(S)** - Method in class sbrt.shell.io.fba.ColumnOrFbaOptOutputFile: Writes the appropriate values in the provided `FbaOptimizationComputer.Results` to this file. **addData(Map<String, Double>)** - Method in class sbrt.shell.io.fba.ColumnOrFbaOptOutputFile: Writes the appropriate values in the provided map to this file. **addData(S)** - Method in class sbrt.shell.io.fba.RowOrFbaOptOutputFile: Writes the appropriate values in the provided `FbaOptimizationComputer.Results` to this file. **addData(Map<String, Double>)** - Method in class sbrt.shell.io.fba.RowOrFbaOptOutputFile: Writes the appropriate values in the provided map to this file. **addData(S)** - Method in class sbrt.shell.io.fba.SerFbaOptOutputFile: Writes the appropriate values in the provided `FbaOptimizationComputer.Results` to this file. **addData(Map<String, Double>)** - Method in class sbrt.shell.io.fba.SerFbaOptOutputFile: Writes the appropriate values in the provided map to this file. **addData(Map<? extends K, ? extends V>)** - Method in interface sbrt.shell.io.MapOutputFile: Writes the appropriate values of the provided map to this file. **addData(S)** - Method in class sbrt.shell.io.MultiOutputFiles: Adds the provided results to the current file. **addData(Map.Entry<? extends K, ? extends V>)** - Method in class sbrt.shell.io.RomoFile: Writes the provided value to this file if the variable is contained in the set returned by `AbstractMapOutputFile.getFilterKeys()`. **addData(Map<? extends K, ? extends V>)** - Method in class sbrt.shell.io.RomoFile: Writes the appropriate values of the provided vector to this file. **addData(S)** - Method in class sbrt.shell.io.SerializedOutputFile: Adds data to this output file. **addData(Map<? extends K, ? extends V>)** - Method in class sbrt.shell.io.SmoFile: Writes the appropriate values of the provided map to this file. **addData(S)** - Method in class sbrt.shell.io.TextOutputFile: Adds data to this output file. **addData(DoubleMatrix<?, String>)** - Method in class sbrt.shell.mng.fba.ExtremeCurrentManager.Rm: Adds data to this results manager. **addedConstraints()** - Method in interface sbrt.kernel.fba.Fluxome: Returns the total number of constraints added to this fluxome. **addedConstraints()** - Method in class sbrt.kernel.fba.IrrevRxnFluxomeV1: Returns the total number of constraints added to this fluxome. **ALGORITHM** - Static variable in class sbrt.shell.mng.AbstractProcessManager: The keyword used to denote an algorithm. **ALGORITHM** - Static variable in class sbrt.shell.mng.fba.RandomFluxVectorManager: The keyword used to denote a random flux vector algorithm. **ALL\_RXNS** - Static variable in class sbrt.shell.text.fba.RxnNameSetFormatV1: The string used to indicate every reaction in a fluxome. **ALWAYS\_ACTIVE\_FILE\_NAME** - Static variable in class sbrt.shell.mng.fba.FluxPlasticityManager: The keyword used to denote the name of the output file for the names of always-active reactions. **alwaysActive()** - Method in class sbrt.kernel.fba.proc.FluxPlasticityProcess.Results: Returns the number of fluxes that are always active. **AMPERSAND** - Static variable in class sbrt.shell.text.SpecialChars: & **AmpersandSetFormat**<E> - Class in sbrt.shell.text: This class is used to define a format for ampersand-delimited sets. **AmpersandSetFormat(SimpleFormat<E>)** - Constructor for class sbrt.shell.text.AmpersandSetFormat: Constructs a new ampersand-delimited set format with the provided element format. **AmpersandSetFormat(Formatter<E>, Parser<E>)** - Constructor for class sbrt.shell.text.AmpersandSetFormat: Constructs a new ampersand-delimited set format with the provided element format. **And**<E> - Class in sbrt.kernel.util: **And(Collection<E>)** - Constructor for class sbrt.kernel.util.And: **AppInputFileLineFormat** - Interface in sbrt.shell.text: This interface is used to represent the format of lines in process input files. **AppInputFileLineFormatVersion1** - Class in sbrt.shell.text: This class is a concrete implementation of `AppInputFileLineFormat`. **AppInputFileLineFormatVersion1()** - Constructor for class sbrt.shell.text.AppInputFileLineFormatVersion1: Constructs a new process input file line format. **ApplicationException** - Exception in sbrt.shell: This class is the superclass of those exceptions that are expected to be thrown from the SBRT shell. **ApplicationException(String)** - Constructor for exception sbrt.shell.ApplicationException: Constructs a new process exception with the provided error message. **ApplicationException(String, Throwable)** - Constructor for exception sbrt.shell.ApplicationException: Constructs a new process exception with the provided error message and cause. **areEquiv(Double, Double)** - Method in class sbrt.kernel.math.DoubleEquiv: Indicates if the provided values are equivalent. **areEquiv(double, double)** - Method in class sbrt.kernel.math.DoubleEquiv: Indicates if the provided values are equivalent. **areEquiv(A, B)** - Method in interface sbrt.kernel.math.EquivTester: Tests the provided objects for equivalence. **areEquiv(Interval, Interval)** - Method in class sbrt.kernel.math.IntervalEquiv: Indicates if the provided intervals are equivalent. **areReqCatalysts(Collection<String>, String)** - Method in interface sbrt.kernel.fba.CatalyzedFluxome: Indicates if the provided collection of catalysts is required for the reaction with the specified name. **areReqCatalysts(Collection<String>, String)** - Method in class sbrt.kernel.fba.IrrevRxnFluxomeV1: Indicates if the provided collection of catalysts is required for the reaction with the specified name. **areRxnNames(Collection<String>)** - Method in interface sbrt.kernel.fba.Fluxome: Indicates if every string in the provided collection is the name of a reaction contained in this fluxome. **areRxnNames(Collection<String>)** - Method in class sbrt.kernel.fba.IrrevRxnFluxomeV1: Indicates if every string in the provided collection is the name of a reaction contained in this fluxome. **areSame(Map<?, Double>, Map<?, Double>, double)** - Method in class sbrt.kernel.math.VectorComparer: **ARROW** - Static variable in class sbrt.shell.text.chem.IrrevRxnFormatV1: The arrow used to signify an irreversible reaction. **ARROW** - Static variable in class sbrt.shell.text.chem.IrrevRxnFormatV2: The arrow used to signify an irreversible reaction. **ARROW** - Static variable in class sbrt.shell.text.chem.RevRxnFormatV1: The arrow used to signify a reversible reaction. **ARROW** - Static variable in class sbrt.shell.text.chem.RevRxnFormatV2: The arrow used to signify a reversible reaction. **ARROW** - Static variable in class sbrt.shell.text.fba.IrrevRxnFormatV1: The arrow used to signify an irreversible reaction. **ASTERICK** - Static variable in class sbrt.shell.text.SpecialChars: \* **asUnmodifiable()** - Method in class sbrt.kernel.util.DiscreteProgressMonitor: Returns this progress monitor as an immutable object. **asUnmodifiable()** - Method in interface sbrt.kernel.util.MutableProgressMonitor: Returns this progress monitor as an immutable object. **asUnmodifiable()** - Method in class sbrt.kernel.util.PercentProgressMonitor: Returns this progress monitor as an immutable object. **AT** - Static variable in class sbrt.shell.text.SpecialChars: `@` **AVG\_P\_FILE\_NAME** - Static variable in class sbrt.shell.mng.fba.SvInitialPointManager: The keyword used to denote the name of the average P output file.

---


|  |  |  |
| --- | --- | --- |
| **Overview** \| Package \| Class \| Use \| **Tree** \| **Deprecated** \| **Index** \| **Help** | |  |
| PREV LETTER   **NEXT LETTER** | **FRAMES**    **NO FRAMES**     **All Classes** |  |


A B C D E F G H I J K L M N O P Q R S T U V W X Y Z 

---
